# Spatiotemporal characteristic of Biantun toponymical landscape for the evolution of Biantun culture in Yunnan, China

**DOI:** 10.1038/s41598-021-03271-2

**Published:** 2021-12-10

**Authors:** Fei Zhao, Jingzhi Cai, Chen Zhang, Guize Luan, Yao Fu, Zhiqiang Xie

**Affiliations:** 1 School of Earth Sciences, Yunnan University, Kunming, 650500 China; 2 State Key Laboratory of Information Engineering in Surveying, Mapping and Remote Sensing, Wuhan University, Wuhan, 430079 China; 3 Beijing Institute of Surveying and Mapping, Beijing, 100038 China

**Keywords:** Socioeconomic scenarios, Sustainability

## Abstract

The geographical environment of Yunnan Province in China and Han migration during the Ming Dynasty contributed to the development of the Biantun culture. Biantun toponyms (BTT) record the integration process between the Central Plains and native Yunnan cultures. The GIS analysis method of toponyms was used in this study to reproduce the settlement characteristics of BTT and the spatial development of the Biantun culture in the Ming and Qing Dynasties. In addition, we have developed a toponymical landscape index to represent the degree of spatial integration between the BTT and ethnic minority toponyms in Yunnan and explore the spatial characteristics of the integration of Han immigrants and local ethnic minorities. The results show that the spatial distribution of the BTT is consistent with the sites selection of the Tuntian (屯田) in Yunnan during the Ming and Qing Dynasties, and the centroids of BTT spread to outskirts and intermontane area from central towns. In the Dali, Kunming, Qujing and other regions, the distribution characteristics of the integrated of BTT and ethnic minority toponyms reflect a higher degree of Sinicization in the central urban areas. Exploring the evolution of Biantun cultural development through the spatial characteristics of toponymical landscapes can help adjust policies for the development and protection of Biantun cultural resources.

## Introduction

The “Tun (屯)” in the Biantun culture originally means cluster, and “Biantun (边屯)” refers to established settlements (clusters) in frontier regions. Specifically, Biantun culture refers to a comprehensive cultural phenomenon that occurred during the historical process of developing, building, and defending the frontier by the ethnic groups who migrated and settled in those frontiers, as well as their native residents. Biantun culture takes the Central Plains culture as the core, relies on the frontier regional culture, integrating the traditional culture of the local ethnic groups, with the typical cultural characteristics of garrison reclamation to guard the frontier^1^.

Yunnan is a large province in the frontier and has been an important region for ethnic migration and cultural exchanges since ancient times. During the Hongwu (洪武) period of the Ming Dynasty, after stabilizing the situation in Yunnan, the Ming government established the WeiSuo (卫所)^2–4^ system and local institutions at different levels, carried out reclamation by garrisoning Yunnan. In order to meet the needs of military defense and facilitate the conditions for farming, the military WeiSuo were mainly stationed at important military places and basin districts, distributed along major traffic roads and around cities and towns^5^. Due to the development of the military Tun, civilian Tun and commercial Tun, a large number of Han immigrants from the Central Plains came to Yunnan and gradually integrated with the local ethnic groups through processes such as intermarriage. With the resulting changes in population structure, ethnic distribution, production relations, political systems, and cultural orientation, the Biantun culture of Yunnan flourished during this period^1^. There are still a large number of Biantun relics in Yunnan, such as toponyms such as Suo (所), Ying (营), Tun (屯), Pu (铺), etc. and guild halls. The protection of Biantun culture in Yunnan Province—including Yongsheng County, the site of Lancang Wei (卫), Mao's Ancestral Hall, and Taliu (他留) culture^6,7^—provides rich resources for modern researchers.

A cultural landscape is an area shaped by historical human activities, which possesses or retain special cultural value^8^. As an important part of a cultural landscape, toponyms are a powerful source of information. Toponyms can preserve information about social, linguistic, and political changes; production economy; military activities; and more^9,10^. As such, toponyms can provide information on the ways in which communities of historical settlers perceived and transformed their environments.

Since 1990, quantitative research has gradually become the mainstream research method of place names. Computer technology has been introduced to optimize traditional research methods, promote the research of place names and cultural landscapes, and accumulate rich research results, such as national toponyms, plant toponyms and so on^11–16^. In addition, Siwei Qian et al.^17^ used spatial analysis methods to study the relationship between the spatial pattern of toponyms, ethnic population and topographical factors. Franco Capra et al.^18^ integrated an ethnopedological approach and mathematical statistics to study Sardinian toponyms influenced by natural and social factors. In summary, the main characteristics of quantitative research on toponyms is to combine traditional cultural geography research ideas, using GIS technology, computer technology and quantitative analysis methods to analyze toponym landscapes^9^.

In this paper, we will analyze the evolution of Biantun culture from the perspective of BTT. The research on the BTT has so far been limited to the review of cultural connotations and historical evolution^1,19,20^. Most studies focused on Yongsheng County^7,21^ and lacked quantitative analysis. From the perspective of historical geography, we use GIS to quantitatively analyze the spatiotemporal distribution characteristics and historical evolution of the cultural landscape of BTT in Yunnan to reveal the relationship between spatial patterns of toponyms and the populations at different periods. The methods used in this study allow exploration of the geographical and spatial patterns of ethnic integration, which may introduce a new perspective on the emergence of Biantun culture.

## Materials and methods

### BTT data

**Figure 1:**
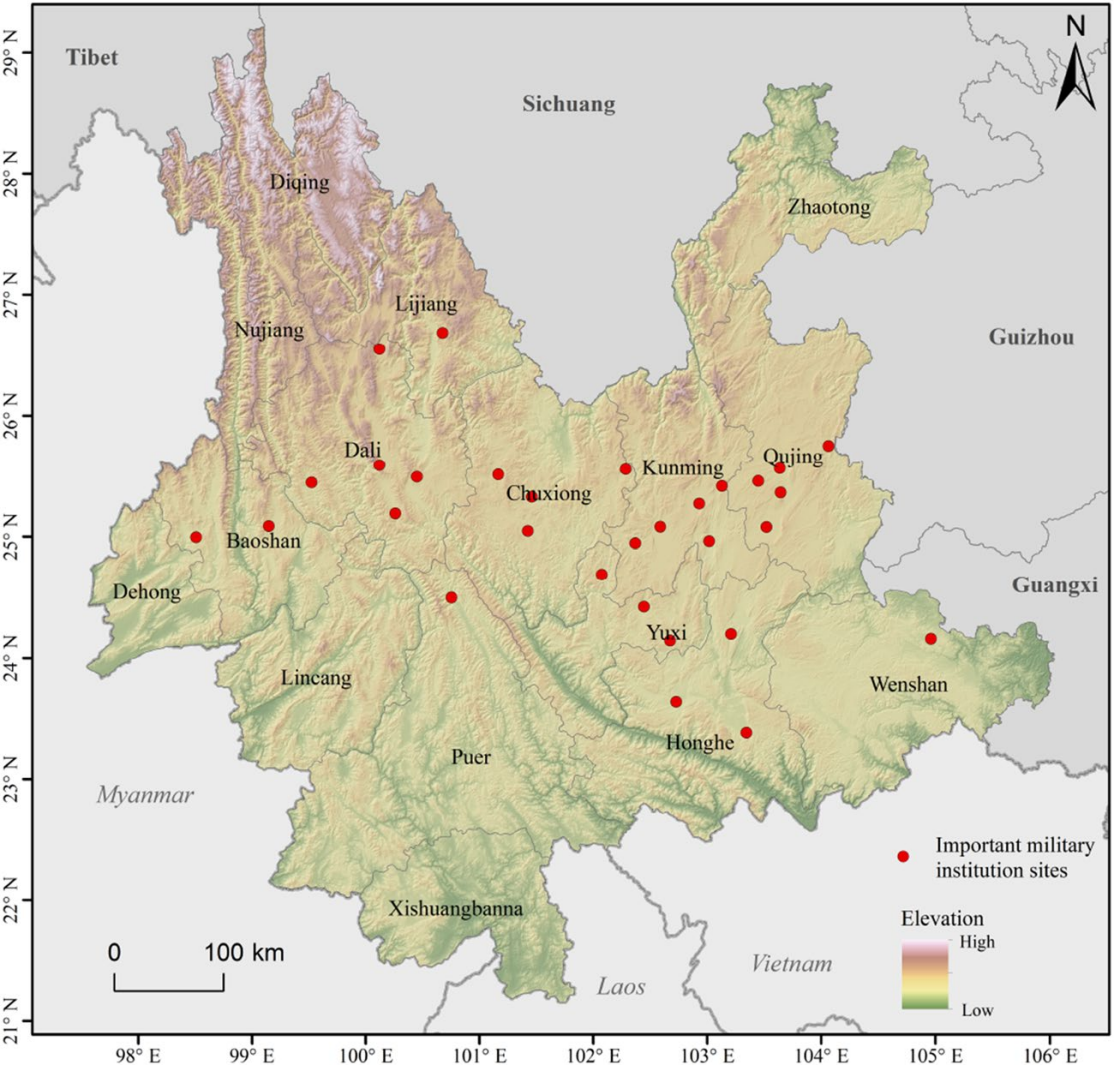
Location of Yunnan and important sites of WeiSuo military institution in the Ming Dynasty. The map was created in ArcGIS Pro v.2.7: https://www.esri.com (Esri, California, USA). The final layout was created in ArcGIS Pro v.2.7: https://www.esri.com (Esri, California, USA).

Yunnan is located on the southwestern border of China (Fig. 1). During the early Ming Dynasty (AD1368-1435), wars in Yunnan were frequent, and living conditions were extremely unstable. During the Hongwu years (AD1368–1398), military immigration of Han ethnic migrants occurred on a large scale^22^. During Yunnan unification and while consolidating the southwest frontier, the institutionalization of land reclamation and crop farming by stationed troops resulted in the mass migration of Han people to Yunnan. Most of the military migrants were concentrated in central towns and Tuntian (屯田) districts, where garrison troops and cultivating grassland/forestland were concentrated in the suburbs of important towns^23^.

According to the Ming Dynasty system, Ying was the basic unit of the army, and Tun was an army resettlement system managed by the metropolis^23^. In the middle Ming Dynasty, conditions in Yunnan began to stabilize. Initially, Han immigrants began settling large-scale settlements on the outskirts of towns, followed by remote areas of flat land. The main Tuntian sites of the middle Ming Dynasty were named based on military units, including Wei, Suo, Qianhu, and Baihu, where the grade hierarchy was Wei (卫) > Suo (所) > Qianhu (千户) > Baihu (百户) ^24^. According to an order from Zhu Yuanzhang (the Ming emperor), the armies of WeiSuo had to be self-sufficient; this required one-seventh of the army to farm and one-third to guard the city^22^. Military yards around the Wei and Suo provided an economic source. Furthermore, the military yards of Yunnan reflected the emergence of toponyms during the middle Ming Dynasty (AD1436–1582), and were named after senior military chiefs’ surnames and suffixed with lower-level transportation trunk facilities and organization; for example, Yi (驿), Bao (堡), Pu (铺), Shao (哨), and others^25,26^.

From the middle to the end of Ming Danasty, along with the development of immigrant agriculture, military yards moved to city outskirts, intermontane basins, and to semi-mountain areas. The original large-scale military yards were not suitable for agricultural development and production under the new terrain, which prompted the original basic organization of the Tuntian to shrink, and small organizational units such as Qi (旗), Wu (武), and Guan to appear. The toponyms that appeared at this time included the surname of the chief of the Qi (旗), Guan (官), and Wu (武), and the lower-level organizational units^27^.

By the end of the Ming and early Qing dynasties (AD1583–1644), conditions in Yunnan were more stable; the population continued to increase and the branches of large clans began to spread. At this time, military-based toponyms faded, and some toponyms added the word “ Cun” (村) after the words used in the original military units. In addition, they followed the floating official system of the Ming Dynasty and the upsurge in commerce, resulting in a large number of settlements wherein the Han nationality merged^5^.

Through the integration of relevant historical materials and documents, thirteen types of place names (Ying (营), Tun (屯), Wei (卫), Suo (所), Baihu (百户), Qianhu (千户), Qi (旗), Guan (官), Wu (武), Yi (驿), Pu (铺), Bao (堡), and Shao (哨)) were selected in this study, with the Ming and Qing dynasties as the research time node. Based on the BTT screening procedure ^28–33^, 1112 toponyms in Yunnan Province were extracted.

The second national toponymic census in China obtained the attribute content of place name origin (placeOrigi) and meaning (placeMeani), historical evolution (placeHisto), geographical entity overview and other attributes through data collation and field investigation. The meanings of toponyms indicate their natural, social, humanistic and economic significance. The historical evolution describes the changes in the creation and modification of toponyms. The information regarding Yunnan rural settlements, urban settlements, and mountain names was obtained from the National Database for Geographical Names of China (http://dmfw.mca.gov.cn/), as presented in Table 1.

**Table 1:** Toponym information of second national toponymic census in China.

Field name	Detail
standardNa	Daying
placeType	Rural settlement
placeOrigi	Named for historical events. Also because of the Liangwang River around the village, the former forest was lush and the scenery was beautiful, it was refined into Huanxiu Village in the early Qing Dynasty, however the folk still call it Daying
placeMeani	The Ming general Mu Ying conquered Queen Liang, and in the 19th year of Hongwu (1386 A.D.), he stationed troops there, and the camp was large
placeHisto	After the 19th year of Hongwu of Ming (1386), Mu Ying's troops had stationed their troops and hoarded supplies here, and because of the large number of troops, they were named Daying
lon	102.827
lat	24.7887

Owing to changes in place names, current place names may not be identified by the aforementioned key words. Therefore, the following steps were used to determine whether the place name was a BTT.

1. Identification of place name attributes.
  Natural language processing integrates disciplines such as cognitive science, linguistics, and computer science to solve problems such as information retrieval, information extraction, and automatic abstraction. As a basic natural language processing technique, Chinese word segmentation recombines consecutive word sequences into word sequences according to certain specifications and performs preliminary attribute processing for screening of BTT ^34,35^. The villages and urban settlements where the stationed troops and military facilities were located during the Ming and Qing Dynasties were judged as BTT and were extracted using keywords such as ‘garrison’, ‘station troops’, and ‘set up sentry post’. The creation date of each toponym was extracted from the historical evolution field. If there is no exact year of the toponym in the field, the Tuntian date is extracted from the two fields of place name meaning and historical evolution.
2. Search historical documents
  If the creation date of toponym and Tuntian date are not known, gazetteers and related documents are checked and the time corresponding to the date of the local historical military immigrants is adopted.

### Research methods

#### Spatiotemporal analysis and visualization in GIS

Mapping the toponyms directly is of limited value; spatial analysis techniques utilizing GIS can readily represent the characteristics of the spatio/temporal distributions of the toponyms, and analyze the effects of military WeiSuo on the development of BTT. Based on the clustering characteristics, the spatial model of the BTT distribution in different periods can be studied using the density characteristics of its points. In this study, the kernel density estimation (KDE) method was used to map the spatial clustering characteristics of all BTT and the small spatial scales of BTT in different periods. KDE is used to calculate the unit density of the point and line elements in a special neighborhood; this method provides the weighted average density of all points in the study area. The principle of KDE is to assign weights according to the distance between a data point and the center point, and the weight increases as the distance from the center point decreases^36^.

**Figure 2:**
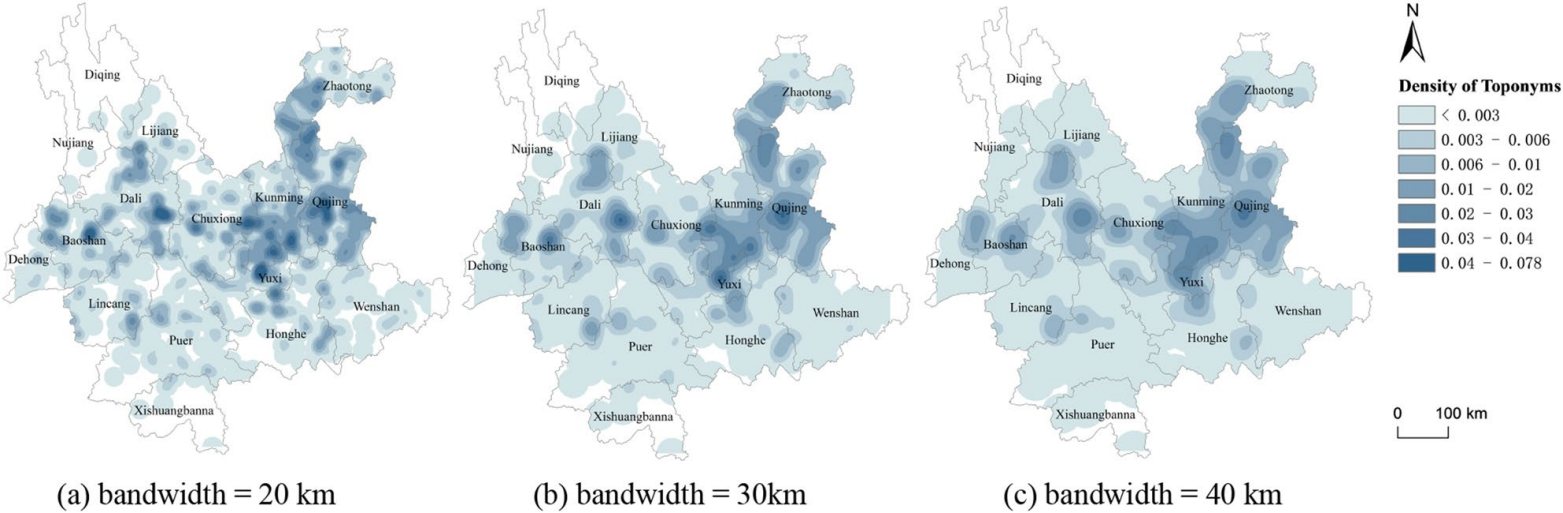
Kernel density of BTT with bandwidth of 20 km, 30 km, 40 km. Maps were created in ArcGIS Pro v.2.7: https://www.esri.com (Esri, California, USA). The final layout was created in ArcGIS Pro v.2.7: https://www.esri.com (Esri, California, USA).

Considering the complicated terrain of Yunnan, we selected a mountain range with large undulations to calculate the average distance between each pair, and finally selected bandwidths of 20 km, 30 km, and 40 km for comparison (Fig. 2) to determine the optimal bandwidth and analyze the distribution characteristics of BTT. It can be observed from the figure that the maximum density value with a bandwidth of 20 km is 0.078, the maximum density value with a bandwidth of 30 km is 0.043, and the maximum density value with a bandwidth of 40 km is 0.034. In KDE, the smaller the bandwidth, the greater is the density value within the bandwidth, and more irregular the distribution of density values; the larger the bandwidth, the smaller is the density value within the bandwidth and the smoother is the density value gradient. With 30 km as the bandwidth, it can clearly identify the density center of BTT and reflect the degree difference of the kernel density of the toponym.

Emerging hot spot analysis (EHSA) examines the clustering of points over time. It uses a space time cube to generate the analysis based on the Getis-Ord Gi* statistic ^37^. A space–time cube creates layers where data is categorized by its x and y coordinates for its geographic location and z coordinates for its year. The space–time cube uses the Mann–Kendall trend test to determine the statistical significance of how the points are changing ^38^. In this study, the input of the time step parameter was adjusted based on this algorithm; therefore, the time domain can be set flexibly.

Large-scale Han military immigrants entered Yunnan in the early Ming Dynasty, but due to frequent wars, there were fewer newly added BTT. As the situation in Yunnan stabilized in the middle Ming Dynasty, a large number of toponyms named after the surnames of upper military officers at Tuntian site were generated, and BTT rose during the period. In the late Ming Dynasty, a considerable number of BTT with names of military establishments such as Guan, Qi, Tun and Bao, and the surnames of lower-level officials were produced. Since the Qing Dynasty, the military characteristics of Han nationality place names have gradually faded^22,24^. According to the above information, the analyses were performed in four time domains: early Ming (1368–1435), middle Ming Dynasty (1436–1582), Late Ming Dynasty (1583–1644), Qing Dynasty (1645–1912).

The standard deviation ellipse (SDE) model is commonly used to analyze spatial distribution characteristics of a point data set, the long and short axes of the ellipse indicate the directions of maximum and minimum diffusion, respectively. The smaller the area, the closer the distribution is to the center of gravity and the more it represents a concentrated distribution. Elliptical deflection angles of 0°/180° and 90° indicate a dominant north–south or east–west direction, respectively^39^. The method was used to reflect the central tendency, dispersion and directional trends in this study.

### Integration index

The integration degree can be interpreted as the degree of pattern difference among the spatial distributions of the BTT and ethnic minority toponyms. We hypothesized that a settlement named by BTT was established by Han migrants. The degree of integration of BTT and ethnic place names calculated in this paper reflects the degree of cohabitation among Han and ethnic minorities and their spatial characteristics.

Based on the cluster structure characteristics of residential areas, the toponym points clustering region can be used as the geographical unit to analyze the distribution characteristics of BTT settlements and ethnic minority settlements. First, extract the overall hierarchical cluster structure by clustering all toponym points, including BTT and ethnic minority toponyms, the distance density constrained clustering method based on Delaunay triangulation is used to extract the point cluster structure in this study^40^. According to the clustering results, the Voronoi regions generated by all the toponym points belonging to the same cluster were merged to obtain the distribution region of each point cluster. After counting the number of BTT and ethnic minority toponyms in each region of each point cluster, we denote *H*_*i*_ as the integration degree of two kinds of toponyms in spatial distribution:

1 $${H}_{i}=\frac{{n}_{e}}{{N}_{i}}-\frac{{n}_{b}}{{N}_{i}}$$

where *N*_*i*_ is the total number of toponyms in point cluster *i*, *n* is the number of ethnic minority toponyms and BTT in the region, *H*_*i*_ ranges from − 1 to 1.

The Voronoi region generated after clustering was used as the geographical unit of index calculation. On the one hand, it can more accurately obtain the influence range of spatial toponymic point elements. On the other hand, compared with the administrative boundary, it can eliminate the influence of non-residential regions.

## Results and discussion

### Spatiotemporal distributional characteristics of the BTT

KDE was performed on all BTT in Yunnan Province. As shown in Fig. 2b, the core (i.e., the highest kernel density distribution) is at the junction of Chuxiong, Kunming, Qujing, and Yuxi. The density center is in the northwest of the junction. The BTT cluster distribution is closely related to the cultivated fields of a garrison of military immigrants of the Ming Dynasty. As the core of military and political affairs of Yunnan, central Yunnan became a region of strong defense and development during the Ming Dynasty. However, western Yunnan, where the migration distribution is the most extensive, has a polycentric characteristic. In eastern Yunnan, the BTT were concentrated in Qujing, which is consistent with the strategic deployment to conquer Yunnan in the early Ming Dynasty^23^. The main traffic line from Yunnan to the interior is located in northeastern Yunnan, the result that the area contains the inner core Han settlements comfirms this situation.

**Figure 3:**
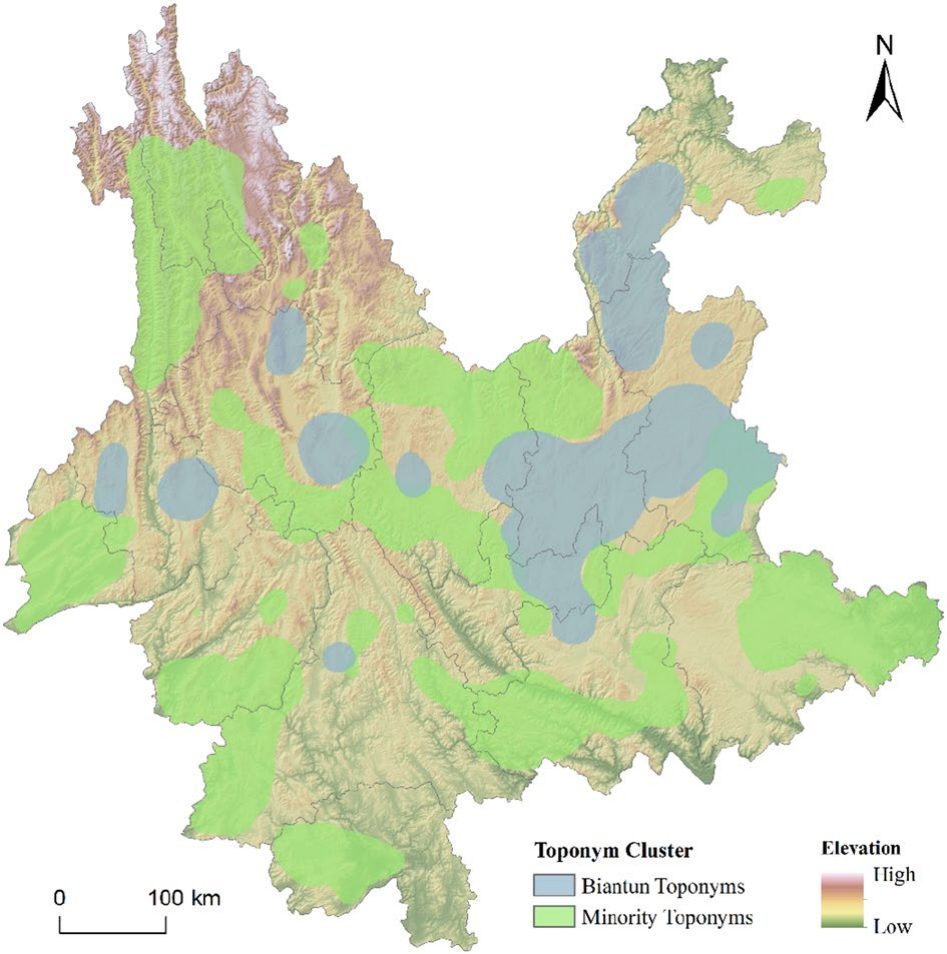
Cluster regions of Biantun toponyms (BTT) and minority toponyms. The map was created in ArcGIS Pro v.2.7: https://www.esri.com (Esri, California, USA). The final layout was created in ArcGIS Pro v.2.7: https://www.esri.com (Esri, California, USA).

Based on analysis of the formation conditions of the BTT, the settlement of Han immigrants with a certain scale was the basis for the increase in BTT, particularly in areas that were suitable for agricultural development and production. Owing to their production mode of small-scale peasant economy, local ethnic minorities in Yunnan were mainly distributed in mountainous areas far from towns, thus, there is a clear spatial difference between the BTT and ethnic minority toponym cluster regions. The high value regions of the result of KDE of ethnic minority toponyms were extracted in this study. Compared with the distribution of BTT (Fig. 3), there are obvious differences in the distribution of the two types of toponyms. The cluster regions of BTT are mostly distributed in the basins of the central, eastern, western, and northeastern regions; ethnic minority toponyms mainly occur in marginal areas, and mostly in hilly and mountainous areas. The results are consistent with the literature and history records^22^.

**Figure 4:**
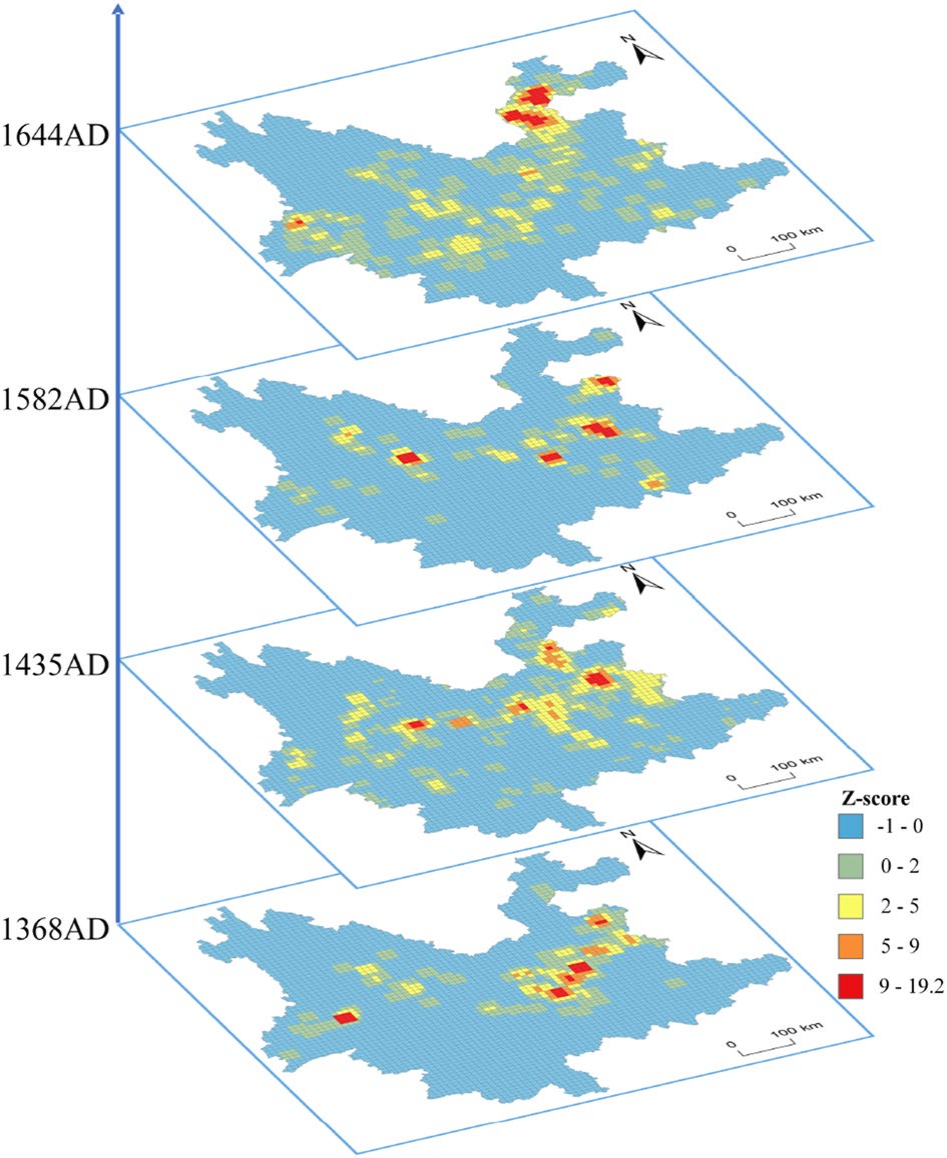
Emerging hot spot analysis z-score. Maps were created in ArcGIS Pro v.2.7: https://www.esri.com (Esri, California, USA). The final layout was created in CorelDraw v. 20.1.0.707 (2018 Corel Corp.).

The Getis-Ord-Gi* statistic takes in attribute values and spatial weights in order to determine statistical significance with a z-score. This z-score is used to determine where high or low concentrations of values are spatially clustered. For statistically significant positive z-scores, the larger the z-score, the more intense the clustering of high values (hot spot). For statistically significant negative z-scores, the smaller the z-score, the more intense the clustering of low values (cold spot). Figure 4 shows the results of the EHSA Z_score (hot spot) in the four periods of BTT. The red area is the hot spot area and the area where the BTT concentrate.

**Figure 5:**
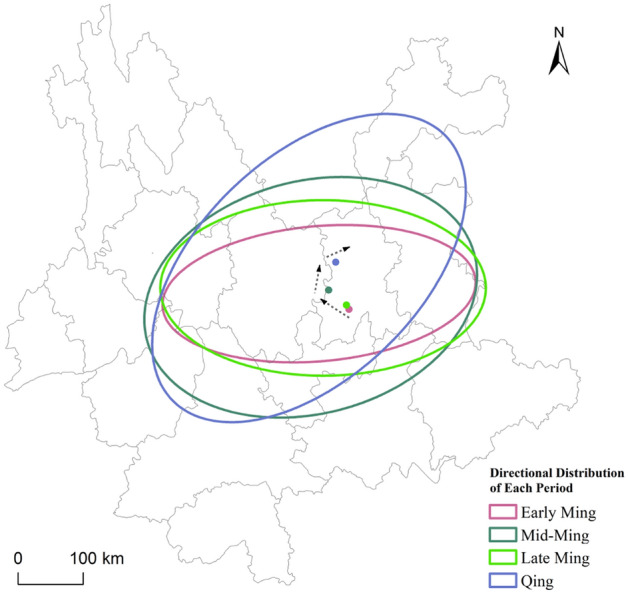
Directional distribution of Biantun toponyms (BTT) in the four periods, arrows indicate the direction change of the distribution of toponyms. The map was created in ArcGIS Pro v.2.7: https://www.esri.com (Esri, California, USA). The final layout was created in ArcGIS Pro v.2.7: https://www.esri.com (Esri, California, USA).

Figure 5 shows the SDE of BTT points in each period. Although place names found in earlier eras still exist in later eras, the calculation of the geometric center of each era was based on new place names to analyze the distribution characteristics of new place names in each era more clearly.

From Figs. 4 and 5, the clustering center of place names for the four periods are distributed in the west of Kunming, which is center of Yunnan Province. Compared with the early Ming Dynasty, the distributions of place names in the next three periods show further expansion. The BTT distribution evolved from the center of Yunnan Province to surrounding areas over time, gradually expanding from central towns and integrating with indigenous peoples.

From the early to the middle Ming Dynasty, the distribution of place names was from the southwest to the northeast. In the middle and late period of the Ming Dynasty, the clustering center of toponyms developed to the southeast, and the distribution direction was west to east. From the middle to the late Ming Dynasty, there is no significant change in the distribution of the clustering center of toponyms and the distribution direction showed east–west horizontal development. By the Qing Dynasty, the distribution direction had clearly developed to the northeast. The BTT in each period differed significantly, indicating that the borderland policies in Yunnan Province differed in the four periods.

Historians believe that the development of BTT in Yunnan originated from the military policy of Tuntian in the Ming Dynasty, and the distribution of BTT is closely related to the location of military WeiSuo^5^. We generated the 30 km buffer zone of the important WeiSuo sites established in the Ming Dynasty, the radius distance of the buffer is consistent with the bandwidth of KDE. The percentage of BTT in the buffer zone is taken to indicate the influence of the WeiSuo sites on the distribution of BTT. BTT adjacent to the WeiSuo account for 52% of all BTT in Yunnan, and they account for 61.1% in the prefectures with WeiSuo sites. These prefectures are mainly distributed in western, central and eastern Yunnan, where heavy troops were deployed in the Ming Dynasty.

In order to study the influence of the WeiSuo system on the naming of BTT, the BTT named after the garrison officer’s surname and the military organization’s function were identified. Such as Xujiaxing (许家营), the commander of the garrisoned here in the Ming Dynasty was surnamed Xu, and Pinanshao (平安哨), it was a institution located on the main traffic line for garrison defense and communication in the Ming Dynasty. There are 14 Biantun toponyms with both attributes, Table 2 shows the numbers and percentages of BTT with different naming methods.

**Table 2:** BTT with different naming methods.

	Officers’ last names	Institutional functions
No	403	132
%	36.2	11.9

### Spatiotemporal evolutionary characteristics of BTT and Biantun cultural landscape

**Figure 6:**
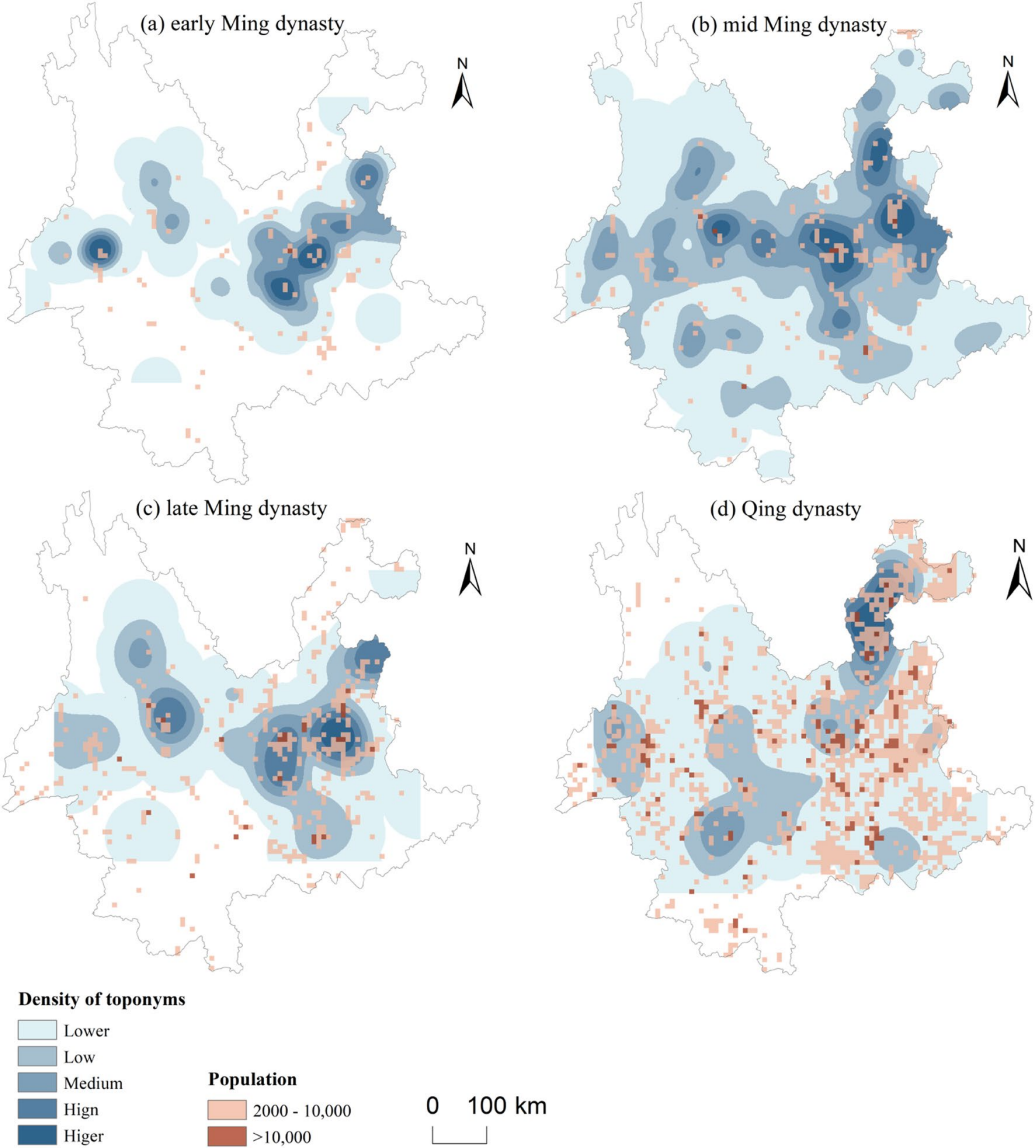
Spatiotemporal evolution of Biantun toponyms (BTT) and population in Yunnan Province. Maps were created in ArcGIS Pro v.2.7: https://www.esri.com (Esri, California, USA). The final layout was created in ArcGIS Pro v.2.7: https://www.esri.com (Esri, California, USA).

Figure 6 shows the spatiotemporal changes in population during each dynasty. Maps show the number of people in a 9 km × 9 km grid cell, obtained from the History Database of the Global Environment (HYDE: https://www.pbl.nl/en/image/links/hyde). The low population in the early Ming dynasty may reflect the unstable situation in Yunnan, which may have made it difficult to count the actual population. In the middle Ming Dynasty, there was a large increase and expansion in both the number of people and spatial distribution, which was possibly caused by a delay in the population statistics relative to the actual population.

As shown in Fig. 6, the population density of central, western, and northeastern Yunnan increased significantly from the early to the middle Ming Dynasty, which is consistent with the large number of military immigrants moving to Yunnan. The distributional area is also consistent with the garrisons established in the early period of the Ming dynasty. In the middle and late period of the Ming Dynasty, as Han immigrants entering Yunnan formed stable settlements, the population density increased significantly, and the distribution range expanded from the center to ethnic minority areas. The integration of various ethnic groups in frontier areas during the Qing Dynasty was strengthened compared with the previous generation. Many Han migrants expanded their families in Yunnan. The official system and devolopment of Shangtun (商屯)^1^ also significantly increased the number of immigrants who moved to Yunnan.

The highest density of BTT was mainly distributed in the Qujing–Kunming–Yuxi area during the early Ming Dynasty. The policy of Tuntian began first in the hinterlands of central Yunnan; this area also became the earliest named area of the BTT. In the middle period of the Ming Dynasty, the BTT distribution expanded to western, northeastern, and southeastern Yunnan and extended from the outskirts of towns to further afield. Various regulations were established in this era. The immigration settlement area was essentially formed and most BTT were derived and named during this period. In the late period of the Ming Dynasty, there were no significant changes in the expansion of BTT. It was common to use the surname of the chief military officer for the names of newly added Tuntian sites at lower levels.

By the Qing Dynasty, the distribution had developed towards intermontane basins, semi-mountainous, and mountainous areas. There were many uprisings and anti-aggression struggles during Qing rule and a considerable number of military immigrants remained in Yunnan.

Based on the spatiotemporal distribution of population and BTT in each period, the evolutional relation between Han migration and the BTT was established. The expansion and evolution of distribution in space and time were largely consistent. The large numbers of Ming Dynasty Han immigrants who moved to the remote regions resulted in greater prosperity Biantun culture, as reflected in the place names. The evolution of the spatial distribution of toponyms also evolved with the development in Tuntian regions: from the central hinterland of Yunnan, it gradually extended away to marginal mountain areas and penetrated into ethnic minority areas.

### Integration between the BTT and ethnic minority toponym

**Figure 7:**
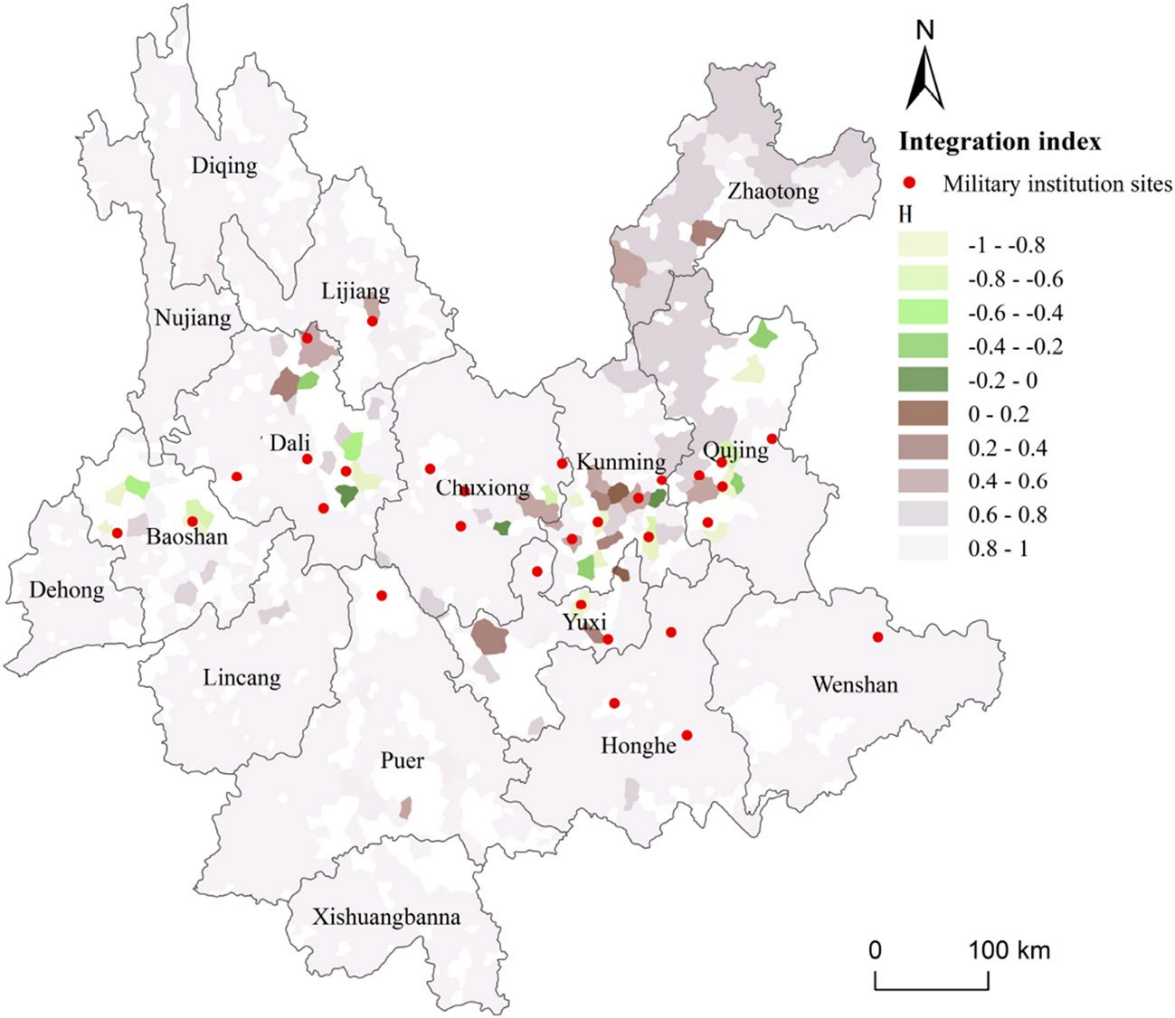
Spatialization results of integration index(Hi), blank areas are areas where toponyms are distributed discretely. The map was created in ArcGIS Pro v.2.7: https://www.esri.com (Esri, California, USA). The final layout was created in ArcGIS Pro v.2.7: https://www.esri.com (Esri, California, USA).

BTT are spread around the important WeiSuo garrisons. We assume that the spatial integration degree of BTT and ethnic minority toponyms is higher in the areas surrounding important WeiSuo garrisons than in the areas far away from WeiSuo, which also reflects the spatial difference in the degree of integration between Han and non-Han ethnicities. Following the integration index Eq. (1), calculate and visualize the integration index of each region of point cluster (Fig. 7). *Hi* > 0 means that there are more ethnic minority toponyms than BTT in the region, and *Hi* > 0 means that there are more BTT than ethnic minority place names in this area. The closer the Hi value is to 0, the closer the number of the two types of toponyms in the region, with greater implied integration of Han and non-Han ethnicities.

The point cluster regions with *Hi* values between 0.6 and − 0.6 mainly occur in Dali and the Chuxiong-Kunming-Qujing area, with further occurrences in Baoshan, Yuxi, and Zhaotong. This is similar to the result of Fig. 2b, which corresponds to the extremely unbalanced settlement distribution of Han immigrants in Yunnan in Ming Dynasty. When Yunnan was suppressed in the early Ming Dynasty, the imperial court took the strategy of occupying the central cities and towns first, conquering Kunming, Dali, Chuxiong, Lin’an, Qujing and other military and political towns, then garrisoned troops to cultivate new arable land. The newly built military and political towns became the most important settlements of Han immigrants in Yunnan in the early Ming Dynasty, and constantly absorbed other kinds of immigrants to settle in the cities^22^. Immigrants are mainly distributed in towns and in basin districts of Tuntian, The Han immigrants who settled in the Fort and Sentry on the traffic road in the remote mountainous area were scattered. From the perspective of the toponymic landscape, there are more cluster regions that are dominated by BTT in Dali, Chuxiong, Kunming, Qujing, and Baoshan (called Jinchi in the Ming Dynasty). Conversely, in Lin'an (Honghe) district non-BTT toponyms are the majority. The deployment of the Ming Dynasty's immigrants to garrison Yunnan is thus reflected in the distribution of geographical names.

Affected by military deployment, the ethnic integration in the central towns and the basin districts will be dominated by Sinicization, while that Han immigrants settled in mountainous and remote areas will integrate into ethnic minorities. The spatial characteristics of the index of toponyms integration degree calculated in this paper show this, and the visualization results show the landscape differences of the integration degree of the two kinds of toponyms.

## Conclusions

Place names are physical carriers and witnesses of the Biantun culture. They retain the process of mutual influence, penetration, and absorption between the cultures of the Central Plains and border region. However, with the development of urbanization and the replacement of place names, some historical place names are facing extinction. This has a significant impact on the protection of traditional landscapes and local historical culture and implies that investigations of the development and historical significance of Biantun culture lack physical elements. Based on GIS spatial analysis, the course of historical development of place names can be intuitively understood, and, in turn, the development of early Han immigration and ethnic integration. The results of this study provide a reference for the protection of toponymical landscape.

As a frontier region, Yunnan represents the origin and development of the Biantun culture. The migration of military Hans during the Ming and Qing dynasties and a large number of commercial immigrants in the Qing Dynasty contributed to the prosperity and development of Biantun culture in Yunnan and the genesis of a large number of BTT. Historically, the BTT began to develop in the early periods of the Ming Dynasty. Yunnan was stable in the middle Ming Dynasty, and a large number of military villages were named. In the later periods of the Ming Dynasty, a small number of names were generated. Spatially, the distribution of the BTT extends from the Kunming–Qujing area to the western, eastern, southwestern, and north-eastern regions of Yunnan, developing from the suburbs of central towns to the intermontane basins and mountainous areas.

National integration is an important prerequisite for the development of Biantun culture. Han immigrants and ethnic minorities lived together and interacted with each other. The Han people intermarried with local ethnic minorities, applied new technologies to agricultural production and water conservancy, established schools, and developed industry and commerce in Yunnan. The resulting integration of Han culture from the Central Plains and the local Yunnan cultures created the distinctive Biantun culture. The integration index proposed in this study measures the integration degree of spatial distribution of BTT and ethnic minority toponyms, and extracts BTT cluster distribution regions, so as to provide spatial data reference for the development and protection of Biantun cultural resources.

Our quantitative analysis of the spatiotemporal distribution and evolution of Yunnan BTT can be used as a basis to study the origin and development of the Biantun culture and to explore the value of Biantun culture and its socio-economic impact on future generations. In addition, it is imperative to discover and protect historical elements (buildings, streets, rivers) and intangible cultural heritage, such as the human landscape and traditional crafts. However, some place names in the study area lack detailed records, and data are based on today's place names; in time, many historic place names have been lost or changed. The screening of BTT can further identify and determine place name descriptions at a semantic level^41^. Further study to link data from social sciences with a GIS approach to classify BTT will improve our knowledge of human influence on the toponymical landscape.
